# Indeterminant Interferon-γ Release Assays in Refugee Children with Splenomegaly, Uganda, 2020–2023

**DOI:** 10.3201/eid3203.251200

**Published:** 2026-03

**Authors:** Christina R. Phares, Moses Mwesigwa, Sean R. Toney

**Affiliations:** Centers for Disease Control and Prevention, Atlanta, Georgia, USA (C.R. Phares, S.R. Toney); Migration Health Division, International Organization for Migration, Geneva, Switzerland (M. Mwesigwa)

**Keywords:** tuberculosis and other mycobacteria, bacteria, respiratory infections, interferon-gamma release assays, splenomegaly, refugee children, refugees, Uganda

## Abstract

We observed a novel association between splenomegaly and indeterminate interferon-γ release assays in refugee children 5–14 years of age in Uganda. Those demonstrating splenomegaly were 4 times more likely to have indeterminate results. Among refugee children 2–4 years of age, >10% had indeterminate results even without splenomegaly.

Refugees bound for the United States must undergo a health assessment before US entry; results are recorded in the Centers for Disease Control and Prevention (CDC) Electronic Disease Notification (EDN) system. In East Africa and other areas with a high burden of tuberculosis (TB), the assessment includes an interferon-γ release assay (IGRA) to detect infection (*1*). Before October 2024, the IGRA requirement applied to applicants 2–14 years of age living in countries with a World Health Organization (WHO)–estimated TB incidence rate ≥20 cases/100,000 population. This requirement has since been expanded to applicants ≥2 years of age. A 2023 CDC review of EDN data for refugees 2–14 years of age noted the proportion of indeterminate IGRA results in Uganda exceeded the expected frequency of ≤2.5% for the QuantiFERON-TB Gold Plus (QIAGEN, https://www.qiagen.com) package insert. 

Splenomegaly is a clinical concern among refugees in Uganda. In a 2015 investigation of 987 US-bound Congolese refugees examined in Uganda, nearly 15% had splenomegaly (*2*). Although the investigation did not establish a definitive etiology, hyperreactive malarial splenomegaly syndrome is a leading cause of massive splenomegaly in malaria-endemic countries (*3*). Prompted by those findings, panel physicians added an enhanced abdominal examination to predeparture health assessments in Uganda that included physical examination and, for palpable spleens, an ultrasound. The abdominal examination occurs before presumptive treatments for malaria and intestinal parasites, which are routinely provided to refugees in Uganda (*4*). Splenomegaly is defined as spleen size by ultrasound >2 SD above the height-adjusted mean. Factors affecting the immune system have been linked to indeterminate IGRA results (*5*,*6*), and splenomegaly can be linked to immune dysregulation. Therefore, we analyzed health assessment data to determine whether splenomegaly was associated with indeterminate IGRA results among refugee children in Uganda. 

Overall, 11,721 refugees 2–14 years of age who were examined in East Africa arrived in the United States during fiscal years 2020–2023 (October 2019–September 2023). Among 1,863 mostly Congolese (86.8%) children examined in Uganda, 87.5% of IGRA test results were negative, 5.7% were positive, and 6.8% were indeterminate. In comparison, among 9,858 mostly Congolese (93.0%) children examined elsewhere in East Africa, 2.9% had an indeterminate IGRA.

In Uganda, the proportion of indeterminate results among older children (5–14 years) was 17.0% for those with splenomegaly and 4.2% for those without splenomegaly; among younger children (2–4 years), proportions were 16.7% and 10.6% (Table). We also modeled the relative prevalence of indeterminate IGRA results by splenomegaly status (Appendix) and found splenomegaly was associated with a significantly increased prevalence of indeterminate results for older children but not younger children. 

**Table:** Interferon-γ release assay result by splenomegaly status by age group among US-bound refugee children examined in Uganda, 2020–2023*

Age group and splenomegaly status	Assay result, no. (%)		Prevalence ratio (95% CI)
	Indeterminate, n = 126	Not indeterminate, n = 1,737	
Age 2–14 y, N = 1,863			
Splenomegaly, n = 177	30 (16.9)	147 (83.1)	3.0 (2.0–4.1)
No splenomegaly, n = 1,686	96 (5.7)	1,590 (94.3)	NA
Age 2–4 y, n = 419			
Splenomegaly, n = 24	4 (16.7)	20 (83.3)	1.6 (0.6–4.0)
No splenomegaly, n = 395	42 (10.6)	353 (89.4)	NA
Age 5–14 y, n = 1,444			
Splenomegaly, n = 153	26 (17.0)	127 (83.0)	4.1 (2.6–6.3)
No splenomegaly, n = 1,291	54 (4.2)	1,237 (95.8)	NA

*NA, not applicable.

Results for repeated IGRAs performed in the United States, typically within 3 months of arrival, were available in EDN for 60 children. Four (7.4%) of 54 older children and 0 of 6 younger children had an indeterminate or borderline result in the United States. US data are limited because results from domestic examination are only captured by EDN for refugees assigned an overseas TB classification. Among the 1,863 refugee children examined in Uganda during the period we studied, 1,754 lacked such a classification, including all 126 children with an indeterminate IGRA result overseas. In addition, a repeat domestic IGRA test is not recommended for those with a positive prior IGRA or with a recent (<6 months) negative IGRA and no TB signs or symptoms (*7*). However, retesting still occurs in practice, as in the case of the 60 children retested in the context of this study.

Our findings suggest a previously unrecognized correlation between splenomegaly and indeterminate IGRA results for refugee children 5–14 years of age in Uganda. Interpreting this correlation is complicated because research has also linked conditions associated with splenomegaly, including malaria, helminthiases, anemia, and HIV infection (*8*), with indeterminate IGRA results (*5*,*6*). Thus, it is unclear whether the observed association is mediated by the pathology causing splenomegaly, impaired splenic function, or other factors. We also noted a high proportion of indeterminate IGRA results for younger children, as others have found (*5*,*6*), even among those without splenomegaly.

The first limitation of this retrospective and programmatic analysis is that the underlying cause of splenomegaly among refugees in Uganda remains uncertain. We lacked systematic data on infections and other clinical factors, limiting inference. IGRA results were qualitative only, preventing assessment of whether indeterminate results related to mitogen control failures or elevated responses in the negative control.

Our observations should be interpreted as hypothesis-generating. Additional investigation is needed to identify the causes of splenomegaly and determine whether the association with increased QFT-Plus indeterminate results reflects splenomegaly itself, its etiologies, comorbidities, or a combination. Further work is also needed to determine whether a link exists for other IGRA tests, other age groups, or other populations with splenomegaly. When a high number of indeterminate IGRA results coincide with prevalent splenomegaly, we advise caution in attributing such results solely to splenomegaly without investigating other potential causes, such as mishandling specimens or errors in processing. This study underscores the prudence of a repeat IGRA after arrival in the United States for any refugee with an indeterminate or borderline IGRA result (*1*).

Appendix Additional information for indeterminant interferon-γ release assays in refugee children with splenomegaly, Uganda, 2020–2023.
