# Supplementary material for: Indeterminant Interferon-γ Release Assays in Refugee Children with Splenomegaly, Uganda, 2020–2023
Source: Emerg Infect Dis. 2026 Mar;32(3):457–9. doi: 10.3201/eid3203.251200 (PMC13016002; doi:10.3201/eid3203.251200)
Supplement: Appendix — Additional information for indeterminant interferon-γ release assays in refugee children with splenomegaly, Uganda, 2020–2023. [file 25-1200-Techapp-s1.pdf]

Article DOI: http://doi.org/10.3201/eid3203.251200

EID cannot ensure accessibility for supplementary materials supplied by authors. Readers who have difficulty accessing supplementary content should contact the authors for assistance.

Indeterminant Interferon-γ Release Assays in Refugee Children with Splenomegaly, Uganda, 2020–2023

Appendix

Additional Methods

To model the relative prevalence of indeterminate IGRA results by splenomegaly status, we used log-binomial regression, stratified by older versus younger children, and fitted additional models that included an indicator for each 2-year age band (among older children) and for wasting as proxy for acute malnutrition (in both age groups). We defined wasting as a Z-score >2 SD below the median weight-for-height for children 2–5 years of age or the median body-mass-index-for-age for children 6–14 years of age using 2009 WHO Child Growth Standards. We excluded from analysis 20 children (1.1%) with missing values or improbable Z-scores (>5 SD above or below the median).

Splenomegaly was associated with a significantly increased prevalence of indeterminate results for older children (prevalence ratio 4.1 [95% CI 2.6–6.3]) but not younger children (prevalence ratio 1.6 [95% CI 0.6–4.0]). Those estimates remained unchanged when adjusted for wasting and age. Neither wasting nor any 2-year age band among older children were associated with indeterminate results.
